# Neuronal oscillations in cognition: Down syndrome as a model of mouse to human translation

**DOI:** 10.1177/10738584241271414

**Published:** 2024-09-24

**Authors:** Pishan Chang, Marta Pérez-González, Jessica Constable, Daniel Bush, Karen Cleverley, Victor L. J. Tybulewicz, Elizabeth M. C. Fisher, Matthew C. Walker

**Affiliations:** 1 Department of Neuromuscular Diseases, UCL Institute of Neurology, London, UK; 2 Department of Clinical and Experimental Epilepsy, UCL Institute of Neurology, London, UK; 3 Department of Neuroscience, Physiology, and Pharmacology, UCL, London, UK; 4 Immune Cell Biology and Down Syndrome Laboratory, The Francis Crick Institute, London, UK; 5 School of Physiology, Pharmacology, and Neuroscience, University of Bristol, Bristol, UK

**Keywords:** Down syndrome, cognition, brain oscillations, abnormal neural activity, translation, dosage-sensitivity gene

## Abstract

Down syndrome (DS), a prevalent cognitive disorder resulting from trisomy of human chromosome 21 (Hsa21), poses a significant global health concern. Affecting approximately 1 in 800 live births worldwide, DS is the leading genetic cause of intellectual disability and a major predisposing factor for early-onset Alzheimer’s dementia. The estimated global population of individuals with DS is 6 million, with increasing prevalence due to advances in DS health care. Global efforts are dedicated to unraveling the mechanisms behind the varied clinical outcomes in DS. Recent studies on DS mouse models reveal disrupted neuronal circuits, providing insights into DS pathologies. Yet, translating these findings to humans faces challenges due to limited systematic electrophysiological analyses directly comparing human and mouse. Additionally, disparities in experimental procedures between the two species pose hurdles to successful translation. This review provides a concise overview of neuronal oscillations in human and rodent cognition. Focusing on recent DS mouse model studies, we highlight disruptions in associated brain function. We discuss various electrophysiological paradigms and suggest avenues for exploring molecular dysfunctions contributing to DS-related cognitive impairments. Deciphering neuronal oscillation intricacies holds promise for targeted therapies to alleviate cognitive disabilities in DS individuals.

## Down syndrome overview

**Figure 1.**
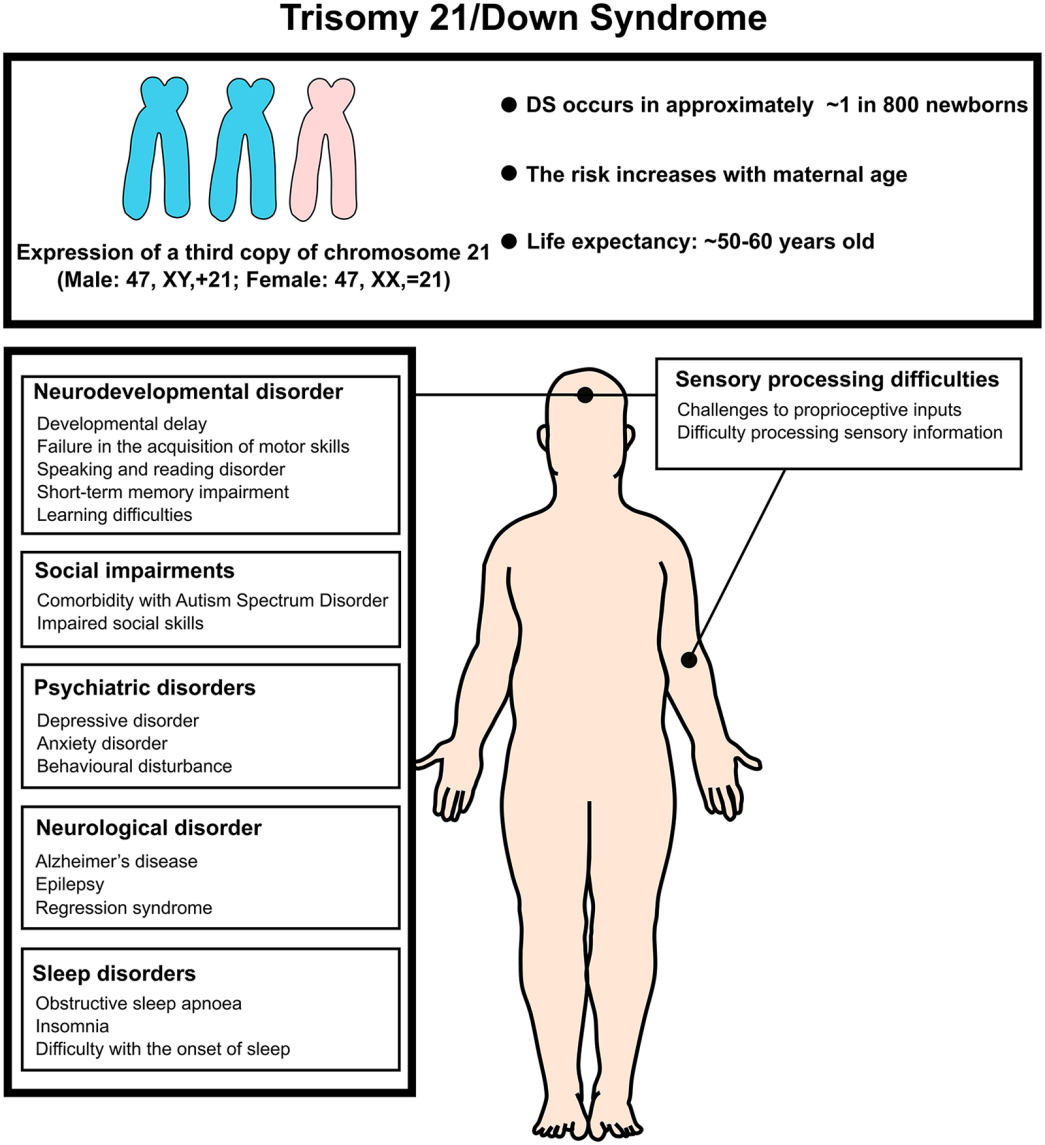
Summary of the abnormal neural condition Down syndrome. Down syndrome, also known as trisomy 21, is the most common autosomal chromosomal irregularity, occurring in approximately 1 in 800 live births. The risk of trisomy 21 increases with maternal age. Most individuals with Down syndrome have full trisomy 21, resulting from meiotic nondisjunction, which produces a genotype with three complete copies of chromosome 21 and a total of 47 chromosomes. Less common forms of Down syndrome include translocation trisomy 21 and mosaic trisomy 21. Clinically, trisomy 21 presents with characteristic facial features, organ malformations (such as heart defects), and abnormal musculoskeletal conditions. It is also associated with an increased risk of neurological disorders and intellectual disabilities.

Down syndrome (DS) is a genetic condition caused by the presence of an additional copy of chromosome 21 (Hsa21), resulting in intellectual disability and many other widespread characteristics that are highly variable across those affected (see Box 1). Most cases (approximately 95%) arise from trisomy 21, while 5% are due to Robertsonian translocation or mosaicism (Wilson and others 2014). The additional copy of chromosome 21 leads to an increased dosage of some gene products, which can disrupt multiple pathways involved in brain development, metabolism, and the formation and operation of intricate neuronal networks that underlie cognitive function (Baburamani and others 2019; Dierssen and others 2020; Weick and others 2013; see Fig. 1). DS occurs in approximately ~12.8 per 10,000 births (~1 in 800 newborns) worldwide (Antonarakis and others 2020; de Graaf and others 2017), and the likelihood of having a child with DS increases with maternal age from ~1 to 12 in 1000 live births for women under 30 years old to ~27 to 100 in 1000 live births for women over 40 years old (Mai and others 2013; Wu and Morris 2013a). Due to advances in health care, individuals with DS now have significantly improved life spans, with the average life expectancy extending into the 50s to 60s in many parts of the world (de Graaf and others 2021; Wu and Morris 2013b). However, the neural mechanisms that link the genotype of DS with its various cognitive phenotypes are still unclear.

Box 1. **Down syndrome.**

Down syndrome (DS) is a genetic disorder caused by total or partial trisomy of human chromosome 21 (Hsa21), that is, by having an extra copy of the genes located on Hsa21. Therefore, it is a disorder of gene dosage, and phenotypes arise from the genes on Hsa21 that are “triplosensitive,” although generally we do not know which genes these are. The incidence of DS is ~1 in 800 births worldwide, and this increases with maternal age.

DS results in a wide-ranging constellation of features whose severity varies extensively between individuals and includes developmental delays, mild to moderate intellectual disability (IQ ranging from 30 to 70), and characteristic physical features. This disorder is also linked to increased risk from certain comorbidities such as congenital heart disease (present in 40% of babies with DS) and early-onset Alzheimer’s disease (AD).

Although DS arises from an increased dosage of certain Hsa21 genes, barely anything is known about the mechanisms underlying the different clinical features of the disorder. Nonetheless, evidence of a role in DS is strong for a handful of Hsa21 genes. For example, the *DYRK1A* gene plays a role in multiple aspects of the disorder such as intellectual disability and congenital heart defects. Similarly, an extra copy of the APP gene has been linked with AD in DS. DS has also been considered a metabolic disorder by many researchers as insulin signaling appears to be altered. However, more research needs to be undertaken to fully elucidate the contribution of Hsa21 genes to DS pathophysiology.

## Neuronal oscillations in the mammalian brain

**Figure 2.**
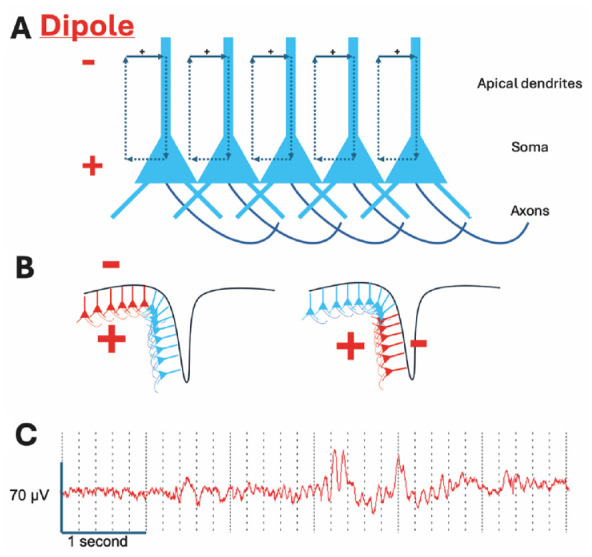
Generation of the electroencephalogram (EEG). (A) The EEG is mainly generated by excitatory input (excitatory postsynaptic potentials) into the apical dendrites of pyramidal neurons, resulting in positive charge flow into the distal dendrites; this generates a dipole with negativity in the region of the distal dendrites and positivity at the soma (see text). (B) The pyramidal neurons are arranged in a laminar fashion, so that activity in gyri generates a dipole with surface negativity (left-hand panel) and activity in sulci generates a tangential dipole (right-hand panel) with lower amplitude negativity and positivity evident at the surface on either side of the source. (C) Six-second sample of scalp EEG from the frontal region demonstrating that EEG is a composite of rhythmic activity at different frequencies. Both slow and faster frequency activity is evident; slower activity tends to be higher amplitude.

The brain is an electrochemical system in which neurons interact by firing action potentials that cause specific neurotransmitters to be released from the synaptic terminal. These neurotransmitters subsequently induce excitatory (i.e., depolarizing) or inhibitory (i.e., hyperpolarizing) currents in their postsynaptic targets by coupling to the corresponding receptors on the postsynaptic membrane. These currents are generated by positively and/or negatively charged ions (cations and anions, respectively) flowing into and/or out of the postsynaptic neuron, creating a potential difference (i.e., voltage) between the intra- and extracellular media. The summation of these voltages across many thousands of neurons generates a local field potential (LFP) in the extracellular medium that can be detected by electrodes placed either on the scalp (the electroencephalogram, or EEG) or directly into the brain (intracranial EEG) (Buzsáki and others 2012) (see Fig. 2).

The detection of LFPs that generate the EEG signal is dependent upon several factors (Jackson and Bolger 2014). First, the neuron consists of a cell body (soma) from which dendrites arise, with the dendritic region being constrained to a specific area. Second, the excitatory input is mainly to the distal dendrites and results in current flow into those dendrites, generating a dipole between the dendrites and the soma. Third, multiple neurons receive the same or similar excitatory input. Fourth, the neurons are arranged in a laminar fashion with a consistent dendritic and somatic region. Excitatory input therefore generates an electrical field with a negative pole in the dendritic region and a positive pole in the somatic region. Coordinated inhibitory input to specific regions of the neuron can also generate a dipole; however, the main charge carriers for inhibition (Cl^–^ and K^+^) have a smaller electrochemical gradient than the charge carriers for excitation (Na^+^ and Ca^2+^) and so the EEG signal is usually dominated by excitatory transmission.

Importantly, the main generator of the EEG signal is the synaptic input to a region rather than the firing of neurons within that region. However, the high amplitude of action potentials and prolonged after-depolarization that they generate allow synchronized spiking in fewer neurons also contribute to the EEG signal. Finally, the primary generators of the EEG signal are layer V pyramidal cells, which receive inputs from both local intracortical and subcortical sources (Thio and Grill 2023). This results in the EEG signal reflecting a combination of top-down and bottom-up processing.

Since the first human EEG recordings reported by Hans Berger in 1924, it has been recognized that the brain can generate oscillations, now recognized as due to rhythmic or quasi-rhythmic voltage fluctuations of ensembles of neurons (see Box 2). The amplitude, location, and frequency of these oscillations were observed to vary according to brain state (e.g., awake or asleep), brain activity (e.g., preparing to move or initiation of movement), and pathology. These oscillations are produced because of the voltage characteristics of neuronal ion channels, the intrinsic resonance of individual neurons and neuronal compartments (e.g., dendrites), reciprocal synaptic connectivity, and the presence and activity of both inhibitory and excitatory synapses. The interplay between these can generate voltage oscillations with frequencies that vary over several orders of magnitude (Buzsáki and Draguhn 2004). This oscillatory behavior has been observed to have a hierarchical structure, and frequency bands of “brain waves” are typically classified as ultraslow (<1 Hz), delta (1–4 Hz), theta (4–8 Hz), alpha (8–12 Hz), beta (12–30 Hz), slow gamma (30–80 Hz), and fast gamma (80–200 Hz; Buzsáki and Draguhn 2004; Plourde and Arseneau 2017; see Table 1). This hierarchical structure ensures that neuronal networks can interact effectively over multiple time scales to support cognitive function (Buzsáki and Draguhn 2004; Jackson and Bolger 2014).

Box 2. **Neural oscillations.**

Oscillatory activity—characterized by periodic fluctuations between different states—is ubiquitous in biological systems, from the beating of a heart to the daily cycle of wake and sleep. Since the first recordings of electrical activity in the human brain by Berger (1929), it has been clear that oscillatory activity is also a hallmark of neural function. Decades of subsequent research have shown that sustained rhythmicity occurs in both population spiking activity and local field potential signals (Buzsaki and Draguhn 2004).

Notably, neural oscillations are not limited to specific species but are conserved across mammals, including rodents, rabbits, bats, dogs, humans, and nonhuman primates (Buzsaki and others 2013). This universality suggests that rhythmic activity serves a functional role in cognition (see Table 1). However, it is important to note that oscillatory activity can also be associated with other brain states, such as loss of consciousness (e.g., during anesthesia), sleep, and pathological conditions like epilepsy.

On a cellular level, empirical evidence indicates that the alternating periods of depolarization and hyperpolarization generated by neural oscillations influence input selection, modulate synaptic plasticity, and coordinate the timing of action potential outputs. As such, neural oscillations are likely to play multiple mechanistic roles in cognition, while abnormal oscillatory signatures are implicated in various aspects of brain dysfunction.

**Table 1.** Focus and Putative Functional Role of Oscillations in Different Frequency Bands.

Name	Frequency Band	Origin	Putative function
Gamma	30–200 Hz	Distributed local networks	Binding, signature of neural activity
Beta	12–30 Hz	Central regions	Movement preparation
Alpha	8–12 Hz	Occipital regions	Attention, sensory processing
Theta	4–8 Hz	Frontotemporal regions	Memory, executive control
Delta	1–4 Hz	Throughout the brain	Sleep, memory consolidation

Gamma band (typically, 30–200 Hz) activity is typically focal and associated with the “binding” of activity in disparate regions to generate compound neural representations (Fries 2009). Moreover, the amplitude of gamma band activity is often modulated by the phase of lower-frequency oscillations (Lisman and Jensen 2013), and this “phase-amplitude coupling” is strongly implicated in memory function (Axmacher and others 2010; Helfrich and others 2018; Heusser and others 2016; Lega and others 2016; Vivekananda and others 2021). Beta band (typically, 12–30 Hz) activity is usually observed in central regions and linked with motor control (Engel and Fries 2010). Alpha band (typically, 8–12 Hz) activity is usually observed in posterior regions and linked with attention and sensory processing (Bagherzadeh and others 2020; Klimesch 2012). Theta band (typically, 4–8 Hz) activity is typically observed in frontal and temporal regions and linked with memory function and executive control (Düzel and others 2010; Eichenbaum 2017; Herweg and others 2020). Finally, delta band (typically, 1–4 Hz) activity is prominent across the brain during slow-wave sleep (Fultz and others 2019) and also associated with memory consolidation (Mölle and Born 2011; Rasch and Born 2013).

Remarkably, despite substantial differences in brain volume, the hierarchical organization of neural oscillations is fairly consistent between mammalian species, including humans, and so rodent models are commonly used in comparative studies (Buzsáki and others 2013). Although initially dismissed as epiphenomena, a growing number of functions have also been ascribed to these oscillations, including representational roles (e.g., encoding the spatial location of an animal in the phase of neural activity; O’Keefe and Recce 1993), attention (directing activity to specific brain areas; Klimesch 2012), communication (synchronizing the activity of disparate brain areas; Fries 2015), memory (encoding, consolidation, and retrieval; Düzel and others 2010; Mölle and Born 2011), and even neuroprotection (facilitating the clearance of toxic metabolites; Fultz and others 2019). However, since changes in connectivity, synapses, and neuronal properties can all affect oscillatory behavior, the association of oscillations with certain functions does not imply causality, and even the disruption of oscillatory behaviors with pathology may be an epiphenomenon rather than a mechanistic explanation of functional deficits.

## The significance of neuronal oscillations for learning and memory

In terms of learning and memory function, theta, gamma, and delta oscillations have been proposed to play a crucial role in mediating the encoding, consolidation, and retrieval of previous experience. Long-term memory is known to be dependent on the hippocampus (Morris and others 1982; Scoville and Milner 1957), and the rodent hippocampus is dominated by theta band oscillations in neural activity during periods of translational movement or arousal (Green and Arduini 1954; Vanderwolf 1969). During learning, these theta band oscillations appear to modulate spike timing (O’Keefe and Recce 1993) and promote rapid synaptic plasticity (Skaggs and others 1996). Disrupting theta band activity in rodents impairs memory function (McNaughton and others 2006; Winson 1978), and increased theta power in the temporal lobes is associated with successful human memory encoding (Herweg and others 2020). During periods of rest or sleep, hippocampal sharp-wave ripple (SWR) events are temporally coordinated with slow-wave oscillations across the cortex and have been proposed to be critical for memory consolidation (Rasch and Born 2013; Staresina and others 2015). Enhancing slow-wave oscillations using auditory (Ngo and others 2013) or direct electrical stimulation (Geva-Sagiv and others 2023) can enhance subsequent memory retrieval, while disrupting SWRs in rodents during periods of rest or sleep after memory encoding impairs memory performance (Girardeau and others 2009).

Finally, increased theta band activity in both the hippocampus and frontal cortex, as well as increased functional connectivity between those regions, is associated with working memory maintenance and long-term memory retrieval in both rodents (Eichenbaum 2017; Jones and Wilson 2005) and humans (Anderson and others 2010; Kaplan and others 2014). The phase coupling (i.e., coordination) of oscillatory activity between disparate cortical regions is believed to facilitate functional interactions by ensuring that neurons are depolarized when inputs arrive (Fell and Axmacher 2011; Fries 2005, 2015). In addition, coupling between the phase of slower oscillations and the amplitude of faster oscillations (phase-amplitude coupling, PAC) is believed to coordinate and maintain sequences of neural activity. Specifically, it has been suggested that cell assemblies encoding for discrete memoranda are active in separate cycles of the higher-frequency oscillation, while sequences of activity across cycles are coordinated by the phase of the lower-frequency oscillation (Lisman and Jensen 2013). PAC has been observed across numerous cortical networks in both humans (Canolty and others 2006) and rodents (Colgin and others 2009), and it is implicated in memory formation (Heusser and others 2016; Lega and others 2016), maintenance (Axmacher and others 2010), consolidation (Helfrich and others 2018), and retrieval (Kaplan and others 2014; Vivekananda and others 2021).

## The significance of neural oscillations for sensory processing

Neural oscillations also play a crucial role in processing incoming sensory signals, and sensory inputs can influence oscillatory activity through mechanisms such as phase resetting, amplitude modulation, and the synchronization of neural populations (Chang and others 2016; Paul and others 2021; Samaha and Postle 2015). These processes are essential for the accurate interpretation and integration of sensory information, modulated by endogenous factors such as arousal, motivation, and top-down control (Bauer and others 2020; Choi and others 2018; Engel and others 2001). Of particular importance to sensory processing is the 8- to 12-Hz alpha rhythm, which is most prominent over the occipital and parietal lobes. Alpha power increases when the eyes are closed during wakefulness and drowsiness, suggesting that it may represent a signature of “cortical idling” (Pfurtscheller and others 1996). In addition, when visual attention is focused on a specific hemifield, alpha power increases over the ipsilateral visual cortex (i.e., in the hemisphere that is not processing the visual stimulus) (Thut and others 2006), and if attention is focused on the auditory component of an audiovisual stimulus, then alpha power increases over the visual cortex (Foxe and others 1998). These results suggest that the alpha rhythm primarily serves to inhibit sensory processing when attention is directed elsewhere (Klimesch 2012). In addition, increased gamma power is associated with the local processing of sensory stimuli, gamma band coherence between disparate regions of the sensory cortex may serve to bind the different stimulus features into a coherent representation (Singer and Gray 1995), and phase-amplitude coupling between alpha and gamma oscillations may serve to prioritize competing sensory representations (Jensen and others 2014).

Historically, neural oscillations related to sensory processing have been regarded as strong predictors of neural dynamics and behavior in both health (Samaha and others 2020) and disease, including age-related disorders such as cognitive decline (Tran and others 2020), neurodevelopmental disorders (Simon and Wallace 2016), and neurodegeneration (Shing and others 2022). Indeed, sensory processing problems are relatively common among individuals with DS (Will and others 2019).

## Abnormal neural oscillations in individuals with DS

Abnormalities in neural oscillations have been observed in EEG recordings from both children and adults with DS, who generally exhibit increased power in lower-frequency bands (i.e., delta, theta, and alpha) compared to typically developing (TD) individuals (see Table 2; Hamburg and others 2021; Smigielska-Kuzia and others 2005; Velikova and others 2011). Increased power in these frequency bands indicates a tighter coordination of neural activity across widespread cortical networks, which can impair information processing and thereby affect learning and memory function (Hanslmayr and others 2012). Indeed, increased theta band power in frontal regions during rest has been shown to correlate negatively with cognitive performance in individuals with DS (Velikova and others 2011). It has also been demonstrated that alpha peak frequency reduces with age in individuals with DS compared to TD adults (Katada and others 2000; Velikova and others 2011). Other studies have shown that individual alpha peak frequency can be a strong (positive) predictor of general cognitive ability (Grandy and others 2013), consistent with reduced alpha peak frequency being associated with intellectual impairment in DS. Finally, people with DS show increased and reduced intra-hemispheric functional connectivity in the delta and alpha bands, respectively (Schmid and others 1992), mirroring similar findings obtained using fMRI (Anderson and others 2013; Pujol and others 2015). Curiously, it has also been demonstrated that the prevalent direction of functional connectivity between occipital regions is from right to left in TD adolescents but left to right in young people with DS—although the implications of this inconsistency are not clear (Babiloni and others 2009).

**Table 2.** Abnormal Neural Oscillations in Down Syndrome (DS) and in DS Mouse Models.

Oscillation	Frequency Range	Human	Mouse Model	Recording State	Increase	Decrease	Reference
Delta	<4 Hz	Adults		Resting state	Power		Babiloni and others 2009; Hamburg and others 2021
	0.5–4 Hz		Dp(16)1Yey	NREM		Power	Levenga and others 2018
Theta	4–8 Hz	Adults		Resting state	Power		Hamburg and others 2021
	6–12 Hz		Dp1Tyb	Spatial memory task		Peak frequency	Chang and others 2020
Alpha	8–9 Hz	Adults		Resting state	Current source density		Velikova and others 2011
	8–9 Hz	Adults		Resting state		Peak frequency	Katada and others 2000; Velikova and others 2011
	10–12 Hz	Adults		Resting state		Current source density	Velikova and others 2011
	8–13 Hz	Adults		Resting state		Power	Babiloni and others 2010; Hamburg and others 2021
	8–13 Hz	Adults		Resting state		Power current density	Babiloni and others 2010
	8–13 Hz	Children		REM		Power	Smigielska-Kuzia and others 2005
	8–12 Hz	Adolescents		Resting state		Power	Babiloni and others 2009
	8.5–14 Hz		Dp(16)1Yey	NREM	Power		Levenga and others 2018
Beta	13–18 Hz	Adults		Resting state	Current source density		Velikova and others 2011
	13–30Hz	Adults		Resting state		Power	Babiloni and others 2009; Hamburg and others 2021
	13–20 Hz	Adults		Resting state		Power current density	Babiloni and others 2010
	13–30 Hz	Adolescents		Resting state		Power	Babiloni and others 2009
	15–30 Hz		Dp(16)1Yey	Wake NREM and REM	Power		Levenga and others 2018
Gamma	30–40 Hz	Adolescents		Resting state		Power	Babiloni and others 2009
Functional connectivity							
Delta	0.4–3.5 Hz	Children and adult		Vigilant state	Coherence		Schmid and others 1992
Theta	4–8 Hz	Children		Relaxed state		Small-world network properties: clustering coefficient	Ahmadlou and others 2013
	6–12 Hz		Dp1Tyb	Spatial memory task	Coherence		Chang and others 2020
Alpha	8–15 Hz	Children		Relaxed state		Small-world network properties: clustering coefficient	Ahmadlou and others 2013
	7.4–12.5 Hz	Children and adult				Coherence	Schmid and others 1992
Global	2–40 Hz	Adolescents		Resting state	Directed transfer function		Babiloni and others 2009
Theta-high gamma	6–12 Hz, 140–160 Hz		Dp1Tyb	Spatial memory task	Coherence		Chang and others 2020
Theta-gamma	6–12 Hz, 60–120 Hz		Dp(10)2Yey	Spatial memory task, open field		Coherence	Chang and others 2020; Muza and others 2023

NREM = non–rapid eye movement sleep; REM = rapid eye movement sleep.

“Human” refers to data from people with Down syndrome (DS); divergent outcomes between mouse and human studies may result from differing data collection methods and cognitive paradigms. Human data come from noninvasive electroencephalogram (EEG) recordings, while rodent studies typically come from invasive EEG recordings. These methodological distinctions are crucial when drawing comparisons, highlighting the need for interdisciplinary collaboration to provide a comprehensive understanding of circadian and cognitive abnormalities in DS.

## Abnormal sleep in individuals with DS

In addition to abnormalities in brain oscillations during wakefulness, oscillatory behavior and brain states in DS are also affected during sleep (see Table 2). Sleep issues, including sleep fragmentation, obstructive sleep apnea, and low sleep efficiency, are common in DS across all ages (Ashworth and others 2013; Edgin and others 2015; Giménez Sandra and others 2018; Lovos and others 2021). Behavioral sleep disturbances affect 52% to 69% of children with DS and 13% to 86% of adults with DS (Esbensen and Hoffman 2017). The broad spectrum of sleep disturbances in adults could be attributed to factors such as the individuals’ age and variations in the definitions and diagnostic methods employed during different studies (Giménez and others 2021). An investigation of EEG changes during sleep in children with DS (1 to 8 years of age) revealed a decrease in alpha power specifically during rapid eye movement (REM) sleep (Smigielska-Kuzia and others 2005). There is a positive correlation between memory retention for object-label associations and the percentage of time spent in REM sleep (Spanò and others 2018), suggesting REM sleep plays a crucial role in memory consolidation and learning processes, and so disruptions in REM sleep may also contribute to the learning difficulties observed in children with DS.

## EEG as a potential biomarker for dementia in individuals with DS

People with DS are at an increased risk of developing Alzheimer’s disease (AD), and the prevalence of AD in people with DS is growing as life expectancy increases (McCarron and others 2017). EEG can be an important tool for identifying AD in adults with DS and potentially for tracking the condition: AD is associated with increased slow-wave activity in the EEG, marked by heightened theta and delta band activity, coupled with heightened complexity of EEG signals (Dauwels and others 2010). Older people with DS have diminished alpha background and are prone to dementia (García-Alba and others 2019). Interestingly, an augmentation in delta and theta wave activity was found within quantitative EEG recordings from people with confirmed DS/AD, in contrast to DS individuals without AD (Salem and others 2015).

## Mouse models of Down syndrome

Down syndrome clinical features arise from abnormal gene dosage, that is, having three copies of dosage-sensitive genes (triplosensitivity). Mouse models of DS, which contain trisomy of all or part of human chromosome 21 or orthologous mouse genomic regions, are providing new insights into the contribution of triplicated genes or groups of genes to the clinical manifestations of DS. However, determining which triplicated genes are responsible for which phenotypes in DS is challenging, as there are >200 protein-coding genes on chromosome 21, most of which are likely not “dosage sensitive/triplosensitive” at least in all tissues. Several approaches are being taken to identify these key “triplosensitive” genes and which human pathologies they cause, including genetic mapping of likely candidate genes in mouse models and validation in human tissues/cell lines (Herault and others 2017; Lana-Elola and others 2016).

**Figure 3.**
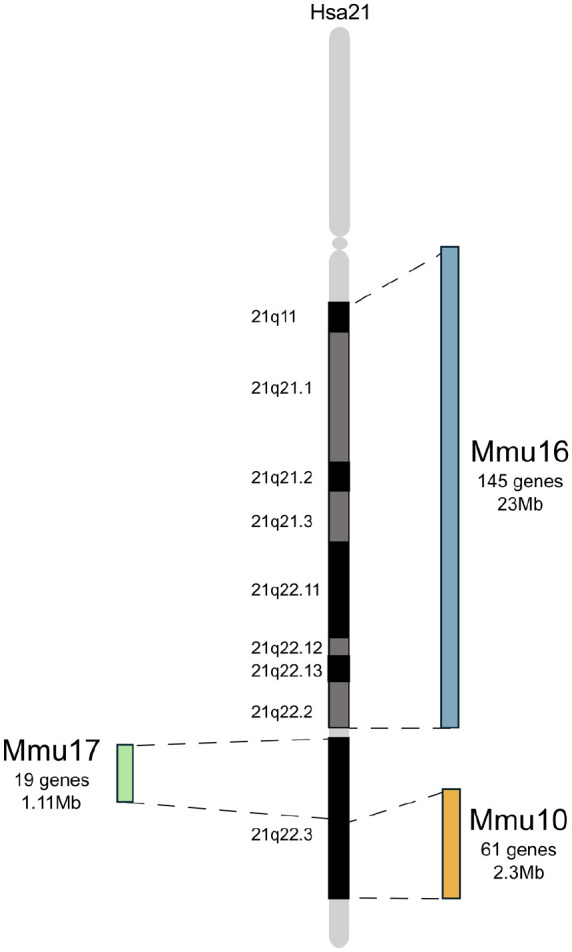
Hsa21 and its orthologous regions in mouse chromosomes 16, 10, and 17. Schematic representation Hsa21 and its associated regions of synteny on mouse chromosome 16 (Mmu16), Mmu17, and Mmu10. Gene numbers for Mmu10 and Mmu17 from Zhang and others (2014) and Mmu16 from Human Genome Assembly GRCh38.

Challenges arise when developing mouse models of DS because Hsa21 orthologous genes are organized into three distinct and separate syntenic regions in the mouse genome, on mouse chromosome 10 (Mmu10), chromosome 16 (Mmu16), and chromosome 17 (Mmu17; see Fig. 3). Nevertheless, many different DS mouse models have been produced, and the refinement of such models has advanced rapidly with the development of chromosome engineering techniques. Thus, all three syntenic regions are available as individual triplications in different mouse strains, mimicking human partial trisomy 21. Yu and colleagues produced the first of these mice and also crossed the strains, such that the Dp(10)2Yey/+;Dp(16)1Yey/+; Dp(17)3Yey/+ (Dp(10)1Yey/+;Dp(16)1Yey/+; Dp(17)1Yey/+) “triple trisomic” model includes triplication of all three Hsa21-syntenic regions on Mmu10, Mmu16, and Mmu17 in the mouse genome (Yu and others 2010).

**Figure 4.**
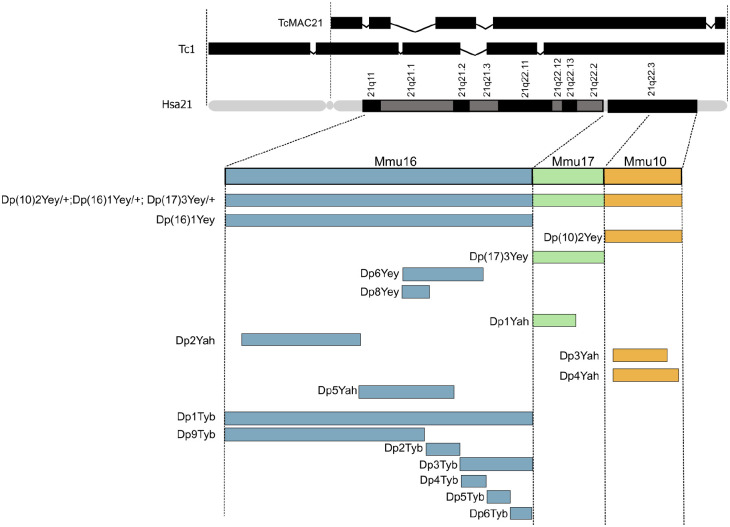
Mouse models of Down syndrome (DS). Hsa21 is shown (q arm with G banding), with triplicated regions in transchromosomic models Tc1 and TcMAC21 depicted above (Kazuki and others 2020; O’Doherty and others 2005) and the syntenic regions of Hsa21 in Mmu16, Mmu17, and Mmu10 shown below, with the triplicated regions contained in each DS mouse model illustrated as bars that span the syntenic regions of Hsa21. Note: the Dp(10)2Yey/+;Dp(16)1Yey/+;Dp(17)3Yey/+ (Dp(10)1Yey/+;Dp(16)1Yey/+;Dp(17)1Yey/+) “triple trisomic” model triplication region spans Mmu16, Mmu17, and Mmu10 (Yu and others 2010). This model was produced by combining triplicated regions in the Dp(16)1Yey (Dp(16Lipi-Zbtb21)1Yey), Dp(10)2Yey (Dp(10Prmt2-Pdxk)2Yey, also known as Dp2Yey) models and Dp(17)3Yey (Dp(17Abcg1-Rrp1b)3Yey, also known as Dp3Yey) (Yu and others 2010). Triplicated regions are broken down further into the strains: Dp6Yey (Dp(16Tiam1-Kcnj6)6Yey, also known as Dp(16)2Yey) (Liu and others 2011) and Dp8Yey (Dp(16Tiam1-Il10rb)8Yey, also known as Dp(16)3Yey) (Liu and others 2014). The Dp1Yah (Dp(17Abcg1-Cbs)1Yah, also known as Ts1Yah) contains a duplicated region of 12 protein-coding genes spanning from *Tff3* to *Cbs* on Mmu17 (Marechal and others 2019). The Dp2Yah (Dp(16Hspa13-App)2Yah) model includes duplication of the region spanning *Samsn1* to *Gabpa* on Mmu16 (Brault and others 2015). The Dp3Yah (Dp(10Cstb-Prmt2)3Yah) model contains a duplicated region spanning from *Rrp1* to the *Pcbp3* gene on Mmu10 (Duchon and others 2008) and the Dp4Yah (Dp(10Cstb-Prmt2)4Yah) and Dp5Yah (Dp(16App-Runx1)5Yah) models are triplicated for other syntenic regions located on Mmu10 and Mmu16 (Duchon and others 2008; Raveau and others 2012). Finally, the Dp1Tyb (Dp(16Lipi-Zbtb21)1TybEmcf) model can be seen, with associated smaller regions of triplication in Dp9Tyb (Dp(16Lipi-Hunk)9TybEmcf), Dp2Tyb (Dp(16Mis18a-Runx1)2TybEmcf), Dp3Tyb (Dp(16Mir802-Zbtb21)3TybEmcf), Dp4Tyb (Dp(16Mir802-Dscr3)4TybEmcf), Dp5Tyb (Dp(16Dyrk1a-B3galt5)5TybEmcf), and Dp6Tyb (Dp(16Igsf5-Zbtb21)6TybEmcf) (Lana-Elola and others 2016).

Hsa21 itself has also been placed into mice, to overcome the issues of having three separate regions of homology in the mouse genome. The Tc1 model contains a freely segregating copy Hsa21 but has notable mosaicism and lacks trisomy of ~50 genes due to internal deletions and chromosomal rearrangements (O’Doherty and others 2005). TcMAC21 was then developed to improve upon this and also contains a freely segregating copy of Hsa21 but with ~93% of protein-coding genes and no detectable mosaicism (Kazuki and others 2020; see Fig. 4).

For several decades, the most widely used mouse model of DS was the Ts65Dn mouse strain, which was generated through an accidental translocation event (i.e., not chromosome engineering), resulting in the presence of three copies of an extra segment from Mmu17, in addition to the translocation of Mmu16 segments onto Mmu17 and Mmu12 (Davisson and others 1990; Reeves and others 1995). This complex rearrangement produced mice that carry most of the region of Mmu16 that has homology to Hsa21 in three copies, but they also have an extra Mmu17 segment that is unrelated to DS, encompassing approximately 35 protein-coding genes, 15 non-protein-coding genes, and 10 pseudogenes that lie on a non-Hsa21 syntenic region of Mmu17—these genes are not triplicated in human DS (Duchon and others 2011; Herault and others 2017). Furthermore, to maintain Ts65Dn mouse colonies, matings are set up between wild-type males and Ts65Dn females (Ts65Dn males are largely infertile), and thus trisomic progeny develop within a trisomic uterine environment, unlike the situation in humans. The presence of the non-DS-related genetic material in the trisomic progeny, along with the trisomic state of the mothers, produces phenotypes that are not related to DS, potentially because several of the irrelevant Mmu17 genes are involved in neurogenesis and neural maintenance (Duchon and others 2022). Hence, results seen in preclinical studies with Ts65Dn mice need to be validated in more accurate models.

As a result of targeted chromosome engineering within the past 15 years, we now have a comprehensive set of mouse strains with duplications of regions of mouse chromosomes orthologous to Hsa21 (Herault and others 2017; see Fig. 4). This mapping panel allows researchers to systematically assign phenotypes, including those related to cognition, to individual regions of duplication and so to potential triplosensitive candidate genes, affording new insights into the underlying gene dosage–related mechanisms of DS (Belichenko and others 2015; Lana-Elola and others 2016). By comparing the phenotypic characteristics of these mouse models with the corresponding human condition, researchers can draw valuable parallels and expand our understanding of DS pathology and potentially drive advances in therapeutic interventions. As the largest region of homology to Hsa21 (145 coding genes) is located on Mmu16, this region has been most extensively studied; the Mmu16 region is triplicated in the Dp(16)1Yey and Dp1Tyb models (Brault and others 2015; Lana-Elola and others 2016; Yu and others 2010; see Fig. 4).

## Abnormal neural oscillations and cognitive function in mouse models of DS

By investigating electrophysiological activity and its relation to cognitive function in mouse models of DS, we can uncover valuable insights into neural dysfunction in people with DS. This comparative approach gives us an understanding of the pathogenic mechanisms of DS and may shed light on potential therapeutic strategies to ameliorate cognitive impairments.

**Figure 5.**
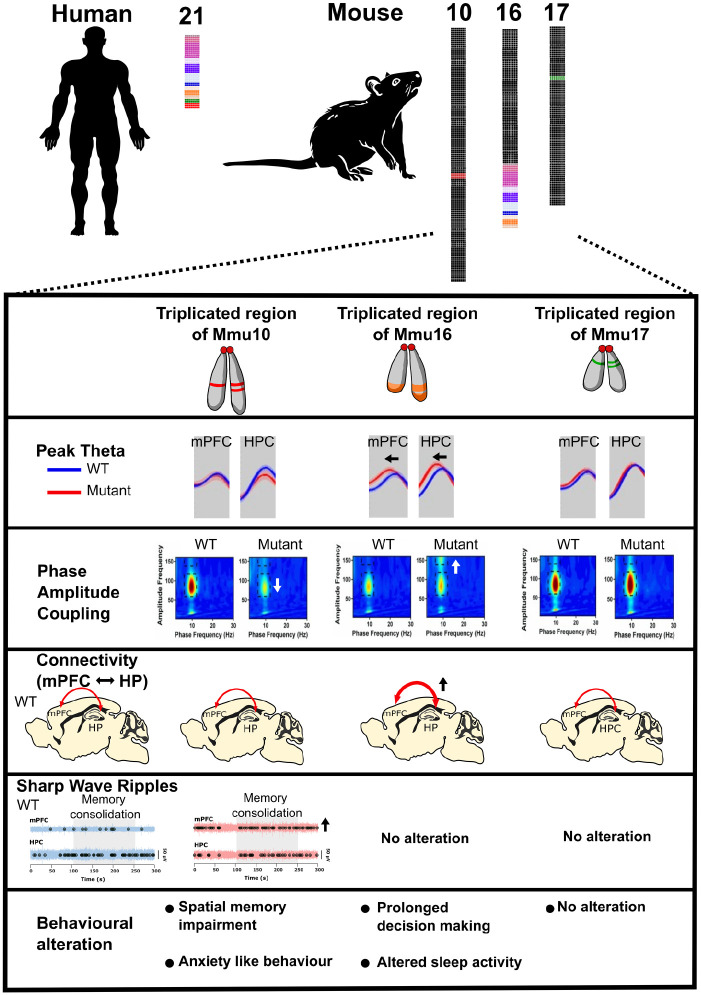
Abnormal neural oscillations and cognitive function in mouse models Down syndrome (DS). Three mouse models of DS carrying an extra copy of the Hsa21-orthologous regions of Mmu10, Mmu16, and Mmu17 offer valuable insights into the role of triplicated genes or gene groups in the clinical manifestations of DS. Three syntenic regions are individually triplicated in various mouse strains, mirroring human partial trisomy. The “triple trisomic” model involves the triplication of all three Hsa21-syntenic regions on Mmu10, Mmu16, and Mmu17 in the mouse genome. In the model with an additional copy of the Hsa21-orthologous region of Mmu10, reduced theta modulation of hippocampal low gamma and increased sharp-wave ripples in the medial prefrontal cortex (mPFC) during memory consolidation periods are observed. Potentially, as a consequence of these changes, the mice exhibit impaired alternation performance and anxiety-like behavior. Similarly, the model with an extra copy of the Hsa21-orthologous region of Mmu16 displays altered theta dynamics, including reduced frequency, increased hippocampal-mPFC coherence, and heightened modulation of hippocampal high gamma. This may be the cause of abnormal cognitive functions, such as prolonged decision-making and disrupted sleep activity. In contrast, neural activity in the mPFC and hippocampus (HPC) in the model with an extra copy of the Hsa21-orthologous region of Mmu17 is not significantly different from the wild type, and these mice do not exhibit abnormal behavior. These results establish a connection between specific hippocampal and mPFC circuit dysfunctions and cognitive deficits in DS models, crucially mapping them to discrete regions of Hsa21.

So, what abnormalities in neural oscillations have been observed in mouse models of DS? First, Dp1Tyb animals—which carry the whole region of homology on the Mmu16 chromosome, around 145 protein-coding genes in triplicate (see Fig. 5)—exhibit delayed decision-making in a spontaneous alternation paradigm, which may be analogous to the delayed reaction times observed in humans with DS (Brunamonti and others 2011), and these delays were associated with reduced peak theta frequency (Chang and others 2020). These results are consistent with human clinical studies that reveal an association between the slowing of brain rhythms and cognitive deterioration, and hence they may serve as a biomarker to predict the onset of dementia (García-Alba and others 2019; Prichep 2007). Dp1Tyb mice also exhibit increased hippocampal theta-gamma phase-amplitude coupling and increased theta phase coupling between the hippocampus and medial prefrontal cortex (mPFC) compared to wild-type littermates (Chang and others 2020; Muza and others 2023). These findings align with clinical observations of heightened low-frequency phase coupling between neural networks in individuals with DS (Anderson and others 2013; Pujol and others 2015). Although enhanced coupling could be viewed as an advantage in some forms of cognitive processing, it could also represent decreased segregation of specialized cortical networks and so could adversely affect information processing. Alternatively, the increased coupling could represent increased “cognitive effort” to solve the task, possibly explaining in the animal model delayed decision-making without an increase in errors. Additionally, there is a deficit in the capability of pyramidal cells to generate bursts and complex spikes, as well as to synchronize during population events in the Dp(16)1Yey model (Raveau and others 2018).

Second, Dp(10)2Yey mice—which have just 37 genes triplicated genes from the Mmu10 region of homology—have notable deficits in spatial memory function, as demonstrated by a reduced tendency to exhibit spontaneous alternation in a T-maze paradigm (Chang and others 2020). In addition, these animals have decreased theta-gamma phase-amplitude coupling in the hippocampus, have an increased occurrence of SWRs in the mPFC during periods of putative memory consolidation, and are more likely to exhibit anxiety-like behavior than their wild-type littermates (Muza and others 2023). Some individuals with DS frequently exhibit signs of low mood, anxiety, decreased interest, and sluggish psychomotor behaviors (Dykens and others 2015). Studies using this mouse model could therefore help to pinpoint distinct patterns of neural activity, triplosensitive genes, and candidate mechanisms that underlie these maladaptive behaviors in humans. In contrast, Dp(17)3Yey mice—which have triplicated genes from the Mmu17 region of homology—did not display any abnormal behavior or neural oscillations in this study (Chang and others 2020), indicating that the genes triplicated in Dp(17)3Yey mice do not play a role these phenotypes (for a summary, see Fig. 5).

Finally, Dp1Tyb mice also have disrupted sleep, including reductions in overall sleep duration and immobility bouts of shorter duration (Lana-Elola and others 2021). Furthermore, the Dp(16)1Yey mice, which bear a duplication of the same Mmu16 genes as Dp1Tyb mice, exhibited altered sleep-related oscillations that closely resemble those observed in people with DS (Fernandez and others 2017; Lovos and others 2021). During the dark phase (night), Dp(16)1Yey mice exhibited increased beta power in wake, NREM, and REM; elevated alpha power during NREM; and reduced delta power during NREM (Levenga and others 2018). Conversely, in the light phase (day), Dp(16)1Yey mice displayed heightened beta power in wake, NREM, and REM; diminished theta power in wake; and decreased delta power during NREM (Levenga and others 2018). Similarly, a quantitative analysis of REM sleep in people with DS showed a reduction of alpha band power in comparison to typically developing individuals (Smigielska-Kuzia and others 2005; see Table 1). Furthermore, when Dp(16)1Yey mice were awake, their theta power was notably reduced, especially during the daytime period of the day/night cycle; this finding aligns with the observation of decreased diurnal and circadian wheel-running activity in these mice (Wong and others 2022). Pronounced changes in EEG oscillations in aged Dp(16)1Yey mice (12- to 14-month-old) have also been recorded, emphasizing distinctive alterations during the dark and light phases. Similarly, individuals with DS consistently exhibit disrupted circadian rhythms, frequently marked by shifts in their daily rhythms (Fernandez and others 2017; Lovos and others 2021; for a summary, see Fig. 5).

As disruptions of circadian rhythms and sleep in DS can have a profound impact on various aspects of neural function, including cognitive and emotional processing, they can directly contribute to poorer functional outcomes in daily life tasks and habits (Churchill and others 2015). Moreover, these disruptions can accelerate cognitive aging and the progression of neurodegenerative diseases, particularly AD and dementia (Cedernaes and others 2017). Indeed, animal studies have shown that age-dependent dysregulation of *RCAN1*, an Hsa21 gene, affects periodicity of the circadian clock, photic entrainment of locomotor patterns, rest-activity profiles, and rhythmicity of activity (Wong and others 2022); it has also been suggested that overexpression of *RCAN1* contributes to the early-age onset of AD-linked pathology in DS (Wong and others 2015, 2022).

## Cellular and molecular mechanisms underlying dysfunction in neural activity and cognition in DS

Triplication of Hsa21 genes produces developmental abnormalities in the brain (Bull 2020), including reduced neurogenesis and defective neuronal maturation that likely contribute to DS cognitive impairment (Stagni and others 2018). Brain function relies on the ability of neurons to communicate with each other, which occurs via electrical and chemical signals (Pereda 2014). This well-orchestrated process underlies behavior—from the simplest execution of motor activities to higher-order brain functions, such as language and memory; as a consequence, even the smallest alterations in any part of the process can have a significant impact (Bartesaghi 2023). For this reason, it is essential to investigate the cellular and molecular mechanisms underlying dysfunction in neural activity in DS mouse models (Chang and others 2020; Muza and others 2023; Raveau and others 2018), with the ultimate goal of identifying causative genes, which can be targeted to try to restore brain wiring and alleviate cognitive defects. Thanks to the latest scientific advances, we can now use human in vitro models, such as induced pluripotent stem cell–derived neurons or organoid-based systems to verify the findings from rodent studies and understand better what is happening in human pathology. The use of those in vitro systems constitutes an important step toward successful translation in drug development (Rowe and Daley 2019).

The disrupted neural oscillations observed in DS individuals and in different mouse models of DS are most likely caused by a combination of morphological alterations in neurons as well as in their neurochemical machinery (Bartesaghi 2023). For example, several studies report that dendrites in DS neurons are atrophic; they are shorter and have a reduced number of dendritic spines compared to dendrites in control neurons. Dendritic atrophy has been linked to dysfunctions of neural networks and intellectual disability in DS and in other neurodevelopmental disorders such as fragile X syndrome and Rett syndrome (Kaufmann and Moser 2000; Nerli and others 2020).

Similarly, the neurochemical machinery involved in neuronal communication appears to be affected in DS. Interestingly, various neurotransmitter and receptor systems have been found altered in DS models, including the glutamatergic (Belichenko and others 2007; Gurjinder Kaur and others 2014), GABAergic (Best and others 2012), and neuropeptide Y systems (Duchon and others 2021), although most of these dysregulations have been found in mouse models and still need to be replicated in humans with DS (Duchon and others 2021). Indeed, the “GABAergic hypothesis” postulates that intellectual disability in DS is caused by an imbalance between excitation and inhibition (Contestabile and others 2017). Specifically, cognitive disabilities in DS have been proposed to be the result of GABAergic overinhibition (Contestabile and others 2017). Although this hypothesis is mainly supported by findings from the Ts65Dn mouse model of DS, whose validity has been extensively questioned by the DS community as it presents triplication of some genes not related to DS, to some extent, this hypothesis is supported in studies of theta-alpha oscillations in adults with DS. Significantly, an association between increased intrinsic self-inhibition within the alpha network and low cognitive ability was found in adults with DS (Hamburg and others 2019). However, this study observed regional differences, indicating that a homogeneous alteration in inhibition is unlikely.

Considerably more investigation is needed to fully understand the cellular and molecular mechanisms underlying abnormal neural activity and cognitive defects in DS. These include logging the neuronal differences in DS mouse models from wild-type littermates and then mapping such phenotypes to individual chromosomal regions, from which candidate triplosensitive genes and pathways can be assessed, ultimately by looking at disruption in human DS cellular models and tissues. Nevertheless, current findings show that, in DS, there are fewer neurons of different subtypes, alterations in glia, and abnormalities of neuronal interconnections, affecting brain function and cognitive performance (Bartesaghi 2023).

## The routes for translation to therapies that help alleviate cognitive impairments in Down syndrome

Currently, there are no pharmacological interventions available to address intellectual disability in DS (Rafii 2022), highlighting the need to comprehend the unique challenges associated with translational research in DS and to devise innovative research approaches (Lee and others 2020).

Based on the fact that several markers of overinhibition, including an increased number of GABAergic interneurons, enhancement of interneuron excitability, and reduced glutamatergic transmission, are altered in the Ts65Dn mouse model of DS (Contestabile and others 2017; Hernández and others 2012; Hernández-González and others 2015), which is in line with the association found between higher general cognitive ability and lower intrinsic self-inhibition in DS individuals (Hamburg and others 2019), drugs aimed at reducing inhibition were proposed as potential therapeutic approaches for cognitive impairment in DS. As expected, treatment with a GABA-_A_-benzodiazepine receptor inverse agonist selective for the α5 subtype was able to improve learning and memory in the Ts65Dn model of DS (Braudeau and others 2011). Those promising results led to the development of a clinical trial aimed at improving cognition in individuals with DS by using an inverse agonist of α5 subunit-containing GABA-_A_ receptors called basmisanil (Roche; ClinicalTrials.gov Identifier: NCT02024789). This phase 2 placebo-controlled trial performed by Roche failed as it was not able to show concomitant improvement of cognition and adaptative functioning of participants aged 12 to 30 years, after six months of treatment (Goeldner and others 2022). This failure is not surprising and can be, at least in part, due to the poor translation between mouse models and humans, especially if therapies have been preclinically assessed in solely the Ts65Dn model of DS that presents triplication of some extra genes that are not related to DS (Duchon and others 2022). In fact, GABA was found decreased in the frontal and temporal cortex of individuals with DS, which, at least a priori, is not in agreement with the GABAergic hypothesis for cognitive impairment in DS (Contestabile and others 2017; Śmigielska-Kuzia and others 2010; Whittle and others 2007).

Changes in oscillatory activity identified by neuroimaging and electrophysiological studies are increasingly being proposed as biomarkers for various neurodevelopmental, neuropsychiatric, and neurodegenerative disorders (da Silva Castanheira and others 2024; Javitt and others 2020). These include altered gamma and theta oscillations in autism spectrum disorder and Rett syndrome (e.g., MECP2 mutations; Guang and others 2018; Wang and others 2013), changes in resting and evoked gamma rhythms in schizophrenia (Schultheis and others 2022), and beta oscillation abnormalities in Parkinson disease and AD (Heideman and others 2020; Monllor and others 2021). Indeed, “oscillotherapeutics” is an emerging field that uses oscillations as both biomarkers and therapeutic targets for disorders associated with brain network dysfunction (Okonogi and Sasaki 2021; Takeuchi and Berényi 2020). In line with this, we hypothesize that DS will exhibit unique oscillatory signatures that can serve as reliable biomarkers. These signatures are expected to reflect the complex genetic landscape of DS and its impact on neural dynamics, potentially showing unique alterations compared to other disorders. A detailed comparison with simpler genetic disorders (e.g., MECP2, SHANK3 mutations) (Rylaarsdam and Guemez-Gamboa 2019) will further advance our understanding of the complexity of DS.

The expression of these biomarkers will likely be modulated by factors such as penetrance and age. For instance, younger individuals with DS might exhibit different oscillatory patterns compared to older individuals, reflecting developmental changes (Cellier and others 2021). Studying the trajectory of oscillatory changes across different age groups in DS will provide information on how these biomarkers evolve over time and how they differ from age-related changes in other disorders. Through this synthesis and hypothesis, a clear and comprehensive framework will be created for understanding how oscillatory activity changes can serve as biomarkers for DS and how these signatures differ from those of other disorders. This approach will enhance the diagnostic and therapeutic potential of oscillatory biomarkers in clinical settings.

Currently, we can conclude that further research is needed to elucidate more precisely the mechanisms underlying cognitive impairment in DS and to identify novel potential therapeutic targets. However, combining gene modulation in mice with the study of neural oscillations seems a promising strategy to achieve successful translation of therapies for cognitive disabilities in DS. It not only enables a precise investigation into the mechanisms underpinning cognitive impairment in DS, facilitating the identification of potential therapeutic targets but also offers avenues for treatment. Approaches like brain stimulation, gene therapy, and pharmacotherapy can be explored, with an emphasis on using “oscillotherapeutics” to address disorders characterized by abnormal neural oscillations, as is the case with DS. The study of brain oscillations in DS could therefore serve as a guide for devising medical treatments and understanding the neural foundations of therapeutic strategies.

## Conclusion

DS, the most common genetic cause of intellectual disability, is increasing in prevalence, so developing an understanding of its underlying mechanisms is paramount. Unraveling the complexities of electrophysiology and circuit dysfunction holds great promise for unlocking crucial insights into the cognitive impairments and developmental challenges faced by affected individuals. By leveraging all our resources, including electrophysiological investigations and accurate mouse models, we can define the molecular, cellular, and circuit-level alterations that contribute to cognitive impairments, helping to identify potential targets for therapeutic interventions and giving greater independence and autonomy to people with DS through effective personalized medicine.
